# Studies on Pd/NiFe_2_O_4_ catalyzed ligand-free Suzuki reaction in aqueous phase: synthesis of biaryls, terphenyls and polyaryls

**DOI:** 10.3762/bjoc.7.41

**Published:** 2011-03-15

**Authors:** Sanjay R Borhade, Suresh Babsaheb Waghmode

**Affiliations:** 1 Department of Chemistry, University of Pune, Ganeshkhind, Pune-411007, India

**Keywords:** heterogeneous catalysis, nickel ferrite, polyaryls, Suzuki reaction, terphenyls

## Abstract

Palladium supported on nickel ferrite (Pd/NiF_2_O_4_) was found to be a highly active catalyst for the Suzuki coupling reaction between various aryl halides and arylboronic acids. The reaction gave excellent yields (70–98%) under ligand free conditions in a 1:1 DMF/H_2_O solvent mixture, in short reaction times (10–60 min). The catalyst could be recovered easily by applying an external magnetic field. The polyaryls were similarly synthesized.

## Introduction

Recently, transition metal catalyzed cross-coupling protocols have generated immense interest owing to their versatile applications in organic synthesis [1–4]. The Pd catalyzed Suzuki reaction between aryl halides with arylboronic acids is one of the most important and powerful methods for the construction of biaryls and polyaryls due to its compatibility towards a wide range of functional groups on both partners [5–10]. The resulting Suzuki products have found numerous applications in the synthesis of natural products, pharmaceutical intermediates, pesticides, advanced materials and liquid crystals [8–10]. These applications, in turn, have led to the production of biaryls and polyaryls on the industrial scale [11]. The Suzuki reaction is usually performed with homogeneous palladium catalysts in the presence of phosphorous ligands. Efforts have been made to enhance the catalytic activity by the use of various ligands containing nitrogen or sulfur, as well as phosphines, salen and *N*-heterocyclic carbenes in traditional organic solvents [12–19]. However, the problems associated with catalyst separation, poisoning, and product contamination, have led to researchers investigating heterogeneous routes [20–21]. In the last few decades, more attention has been given to overcome these problems by employing a number of heterogeneous palladium systems, such as palladium supported on a variety of support materials (e.g., carbon, metal oxides, zeolites, clays, polymers, diatomite and graphite oxide) as well as in ionic liquids [22–32]. In addition, efforts were also made to replace the environmentally harmful organic solvents by eco-friendly solvents such as water to make the scale up procedure economical and viable for industrial applications [33–37]. This has led to the use of water soluble ligands, additives and reusable heterogeneous supported Pd catalysts [38–39]. However, the limited availability of water soluble aryl halides and the limited scope of the Suzuki reaction under ligand free conditions in the absence of additives thus faced with these limitations requires a fresh approach to carry out the Suzuki reaction.

Recently, the use of Pd supported on surface-modified nano NiFe_2_O_4_ catalyst for the Heck and Suzuki reactions was demonstrated [40]. The most important paradigm of this reaction is the easy removal of catalyst from the reaction mixture by employing an external magnetic field. We recently reported the filtration-free magnetically separable Pd/NiFe_2_O_4_ catalyst for the Heck reaction [41]. Herein, we report the successful exploitation of the magnetically recoverable Pd supported on NiFe_2_O_4_ catalyst for the Suzuki reaction with various substrates and the optimization of reaction conditions under ligand free heterogeneous conditions in an aqueous phase. Moreover, to the best of our knowledge, this is the first report describing the synthesis of polyaryls under these reaction conditions and with this catalyst.

## Results and Discussion

Nickel ferrite samples were synthesized as reported earlier [42]. Loading of Pd on nickel ferrite was carried out by wet impregnation and subsequently reduced by the continuous flow of hydrogen gas [43]. The scanning electron microscope image shows that particle size of the catalyst is ~100 to 300 nm (Supporting Information File 1). The X-ray photoemission spectra confirms the formation of metallic Pd(0) particles, the binding energy values were 335.3 and 340.5 eV for Pd(3d_5/2_) and Pd(3d_3/2_) core levels, respectively (Supporting Information File 1). The catalyst is stable under the employed reaction conditions, which was confirmed by the X-ray diffraction pattern of fresh and spent catalyst (Supporting Information File 1). The coupling of iodobenzene with phenylboronic acid was studied as the model reaction in air and the reaction conditions were systematically optimized. The reaction was carried out using Na_2_CO_3_ as the base in the presence of 0.1 mol % Pd at 90 °C.

We initially studied the effect of solvents and the addition of water as the co-solvent on the Suzuki coupling reaction (Table 1). These results indicated that the reaction in polar aprotic solvents such as dimethylformamide (DMF), dimethylacetamide (DMA), *N*-methylpyrrolidin-2-one (NMP) and dimethylsulfoxide (DMSO) showed only 34–50% conversion after 2 h (Table 1, entries 1–4). The reaction in ethereal, alcoholic, and non-polar solvents was sluggish (Table 1, entries 5–10). However, the addition of water as co-solvent to the polar aprotic solvents greatly improved the rate of reaction. The addition of water to DMF led to a very rapid increase in the reaction rate: The reaction being completed after 5 and 10 min when the ratio of DMF and water was 1:1 and 3:2, respectively (Table 1, entries 13–18). The results suggested that, the ratio of water to DMF played an important role in the Pd/NiFe_2_O_4_ catalyzed Suzuki reaction.

**Table 1:** The effect of different solvents on the Suzuki coupling reaction^a^.

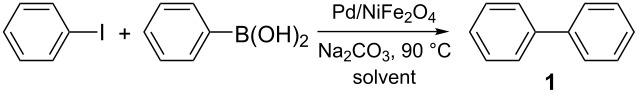			
Entry	Solvent	Time (min)	Conversion (%)^b^

1	DMF	120	50
2	DMA	120	34
3	NMP	120	43
4	DMSO	120	46
5	Diglyme	120	7
6	Xylene	120	2
7	Diphenyl ether	120	3
8	DME	120	6
9	Dioxane	120	10
10	EtOH	120	1
11	H_2_O	120	95
12^c^	H_2_O	120	98
13	DMF/H_2_O (9:1)	30	66
14	DMF/H_2_O (4:1)	30	89
15	DMF/H_2_O (7:3)	30	97
16	DMF/H_2_O (3:2)	10	100
17	DMF/H_2_O (1:1)	5	97
18	DMF/H_2_O (2:3)	5	89
19	Dioxane/H_2_O (1:1)	15	95
20	DME/H_2_O (1:1)	15	100
21	MeCN/H_2_O (1:1)	15	100
22	EtOH/H_2_O (1:1)	60	73

^a^Reaction conditions: iodobenzene (1 mmol), phenylboronic acid (1.2 mmol), Na_2_CO_3_ (2 mmol), Pd/NiFe_2_O_4_ (0.1 mol %) and 4 mL of solvent at 90 °C. ^b^Conversions were determined by GC (∆_rel_ = ±5%). ^c^Tetrabutylammonium bromide was added.

Among the various bases studied, inorganic bases were superior to organic bases such as triethylamine (TEA) and tributylamine (TBA) which may be due to partial inhomogenity in the aqueous phase. As the basicity of alkali carbonate increases, the time for completion of the reaction increases (Table 2, entries 4–6). Na_2_CO_3_ was found to be the best base (Table 2, entry 4). Sodium hydroxide did not appear to be an effective base under these reaction conditions (Table 2, entry 3).

**Table 2:** The effect of various bases on the Suzuki coupling reaction^a^*.*

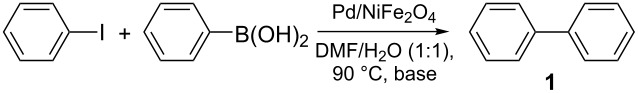			
Entry	Base	Time (min)	Conversion (%)^b^

1	TEA	60	79
2	TBA	60	100
3	NaOH	60	50
4	Na_2_CO_3_	5	97
5	K_2_CO_3_	20	100
6	Cs_2_CO_3_	30	88
7	NaHCO_3_	30	94
8	NaOAc	60	97
9	Ca(OH)_2_	60	79

^a^Reaction conditions: iodobenzene (1 mmol), phenylboronic acid (1.2 mmol), base (2 mmol), Pd/NiFe_2_O_4_ (0.1 mol %) and 4 mL of 1:1 DMF/H_2_O at 90 °C. ^b^Conversions were determined by GC (∆_rel_ = ±5%).

The effect of temperature on the activity of Pd/NiFe_2_O_4_ catalyst was studied in the range 30–100 °C. Table 3 shows that the percentage conversion increased with temperature. The reaction can be performed at room temperature, but the reaction time was significantly increased (Table 3, entry 1).

**Table 3:** The effect of various temperatures on the Suzuki coupling reaction^a^.

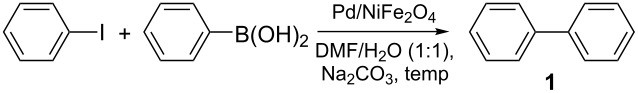			
Entry	Temperature	Time (min)	Conversion (%)^b^

1	30	360	88
2	50	120	86
3	70	60	98
4	80	10	99
5	90	5	97
6	100	5	99

^a^Reaction conditions: iodobenzene (1 mmol), phenylboronic acid (1.2 mmol), base (2 mmol), Pd/NiFe_2_O_4_ (0.1 mol %) and 4 mL of 1:1 DMF/H_2_O at various temperatures. ^b^Conversions were determined by GC (∆_rel_ = ±5%).

The effect of Pd concentration on the Suzuki reaction was also studied. A graph depicting conversion versus time showed that the catalytic activity increased with Pd content (Supporting Information File 1). The reaction was complete within 5 min, with excellent conversion using 0.5 mol % Pd. A decrease in Pd concentration from 0.25 to 0.1 mol % did not influence the rate of reaction to any great extent. However, when the Pd concentration was lowered to 0.005 mol %, the catalyst still functioned very well, but the conversion was too low to be of practical use.

Using the preliminary optimized reaction conditions, we explored the general applicability of Pd/NiFe_2_O_4_ catalyst with various boronic acids and aryl halides containing electron withdrawing or donating substituents (Table 4). The Pd/NiFe_2_O_4_ catalyzed Suzuki reaction tolerated a wide range of functional groups such as NO_2_, CHO, Me, Cl, OMe, OH, NH_2_, and Ac. The aryl iodides and electron deficient aryl bromides showed excellent reactivity with phenyl- and 3-(hydroxymethyl)phenylboronic acid and led to the expected products in high yields within short reaction times. Bromobenzene and electron rich aryl bromides required a higher loading of palladium (1.0 mol %) to give comparable results. Both electron rich and electron deficient arylboronic acids gave biaryl products in good to excellent yields. Heterocyclic boronic acids required longer reaction times compared to arylboronic acids, probably as a result of strong coordination of the heterocyclic nitrogen with Pd metal. Ortho substituted aryl iodides required slightly longer reaction times to give the corresponding biaryls. The coupling of 4-chloroiodobenzene with phenylboronic acid gave exclusively 4-chlorobiphenyl in good yield. However, under these reaction conditions, selective monoarylation of 4-bromoiodobenzene did not occur even with a stoichiometric amount of phenylboronic acid. The comparatively less reactive chlorobenzene did not undergo satisfactory conversion even after 24 h (Table 4, entry 35). Whereas, activated aryl chlorides gave the coupling product in moderate yields after 24 h (Table 4, entries 33, 34).

**Table 4:** Suzuki cross coupling reaction of aryl halides with arylboronic acid^a^.

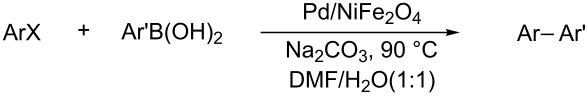					
Entry	Aryl halide	Arylboronic acid	Product	Time (min)	Yield^b^ (%)

1	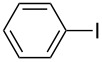	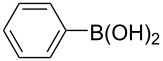	**1**	5	97
2	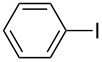	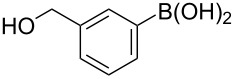	**2**	15	92
3	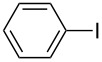	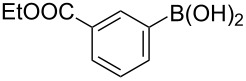	**3**	15	96
4	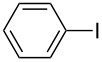	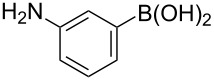	**4**	20	91
5	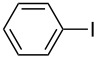	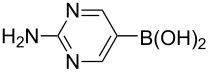	**5**	60	78
6	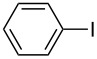	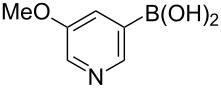	**6**	60	72
7	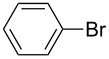	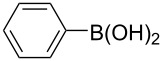	**1**	60	69
8^c^	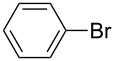	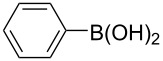	**1**	30	98
9^c^	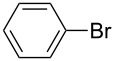	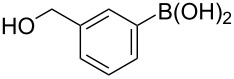	**2**	45	95
10^c^	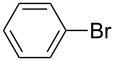	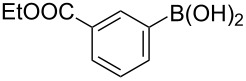	**3**	30	92
11^c^	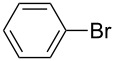	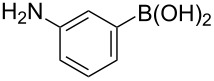	**4**	35	89
12^c^	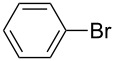	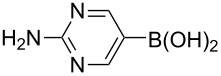	**5**	60	68
13^c^	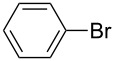	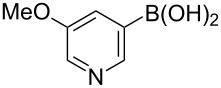	**6**	60	76
14^c^	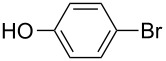	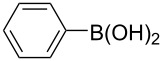	**7**	150	78
15^c^	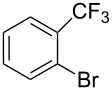	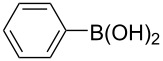	**8**	120	81
16	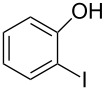	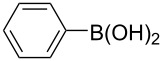	**9**	45	80
17	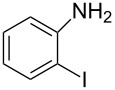	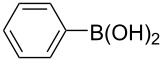	**10**	60	75
18	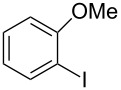	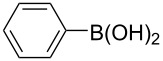	**11**	60	86
19	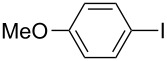	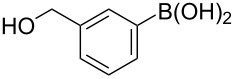	**12**	30	96
20	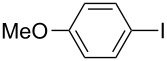	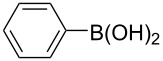	**13**	20	94
21	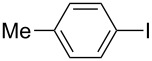	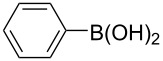	**14**	30	97
22	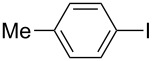	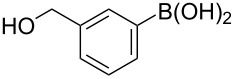	**15**	30	96
23	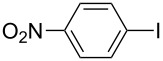	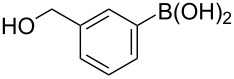	**16**	30	94
24	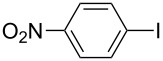	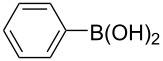	**17**	15	96
25	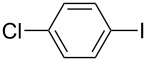	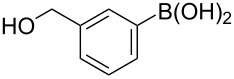	**18**	15	89
26	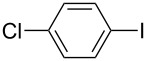	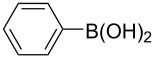	**19**	15	93
27	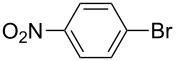	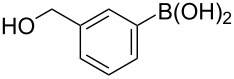	**16**	30	95
28	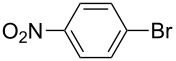	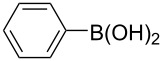	**17**	15	95
29	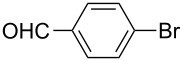	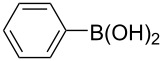	**20**	15	88
30	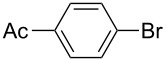	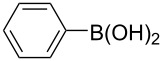	**21**	15	96
31	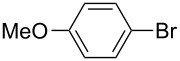	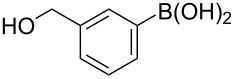	**12**	30	85
32	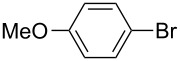	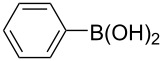	**13**	30	94
33^d^	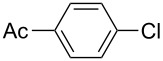	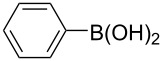	**21**	24	23
34^d^	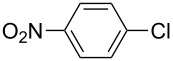	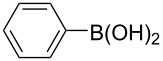	**17**	24	42
35^d^	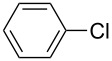	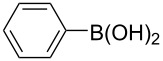	**1**	24	6

^a^Reaction conditions: aryl halide (1 mmol), arylboronic acid (1.2 mmol), base (2 mmol), 4 mL of 1:1 DMF/H_2_O and Pd/NiFe_2_O_4_ (0.1 mol %) at 90 °C. ^b^After purification by flash silica gel chromatography. ^c^1 mol % of Pd/NiFe_2_O_4_ used for entries 8–15. ^d^0.25 mol % of Pd/NiFe_2_O_4_ used at 105 °C and time reported in h for entries 33–35.

Recently, terphenyls have attracted attention due to their wide range of significant biological applications including potent immunosuppressant, neuroprotective, antithrombotic, anticoagulant and cytotoxic activities [44–47]. Polyphenyls are also an important structural element in liquid crystals and fluorescent compounds [48–52]. Terphenyls have been previously synthesized by the reaction of aryl- or benzylzinc reagents with functionalized biphenyl nonaflates [53], Grignard reagents with dihalobenzenes [54–56]: Other methods give poor yields. The Suzuki cross coupling protocol has been used for the synthesis of terphenyls and polyaryls using Pd(OAc)_2_ or Pd(PPh_3_)_4_ with or without ligands in homogeneous medium [57–60]. Although, these authors have reported good yields of terphenyls and polyaryls, the fact that the precious palladium catalyst is non-recoverable renders these processes commercially undesirable. The first use of heterogeneous Pd-modified silica catalyst for consecutive Suzuki reactions was reported by Clark et al. for the synthesis of terphenyls as well as polyaryls using di- and tribromoarenes with phenylboronic acids in *o*-xylene as solvent at 120 °C for 20 h under a N_2_ atmosphere [61]. We have studied the usefulness of Pd/NiFe_2_O_4_ catalyst for the synthesis of terphenyls and polyaryls in a single step under the optimized reaction conditions. The results thus obtained are summarized in Table 5. The reactions were performed by using 1.0 mol % of Pd at 90 °C and 3.5 equiv of arylboronic acid in 1:1 DMF/H_2_O solvent for 2 h. The reaction between various di- and trihalo aryls with phenyl- and 3-(hydroxymethyl)phenylboronic acids led to multiple coupling products in good to excellent yields (Table 5, entries 1–16).

**Table 5:** Suzuki cross coupling reaction of di- and trihaloaryls with arylboronic acid^a^.

Entry	Aryl halide	Arylboronic acid	Product	Yield^b^

1	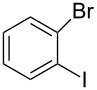	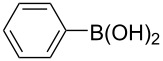	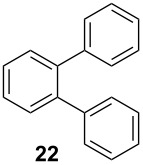	73
2	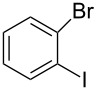	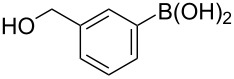	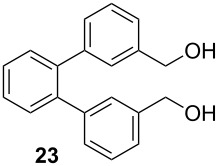	62
3	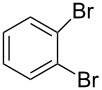	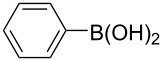	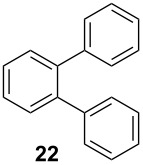	69
4	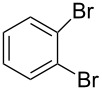	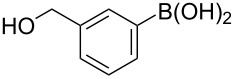	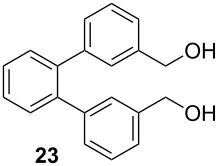	66
5	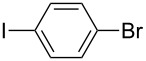	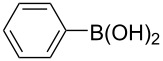	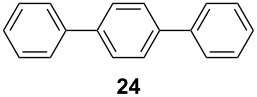	88
6	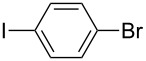	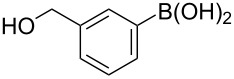	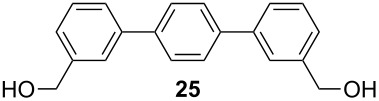	76
7	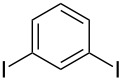	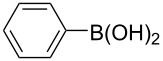	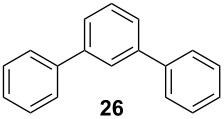	92
8	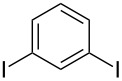	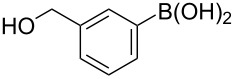	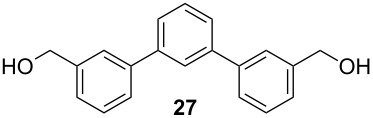	79
9	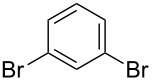	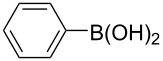	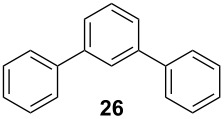	83
10	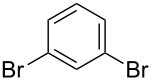	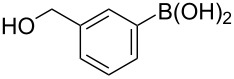	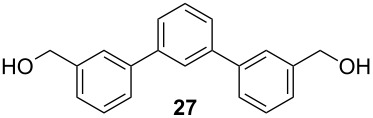	72
11	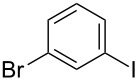	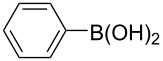	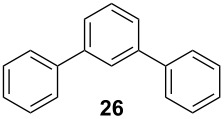	84
12	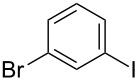	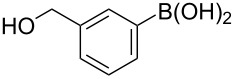	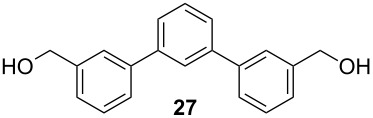	78
13	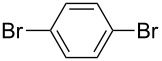	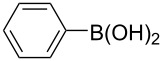	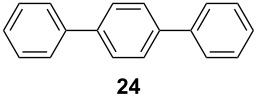	78
14	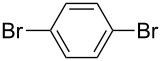	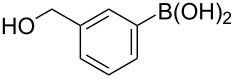	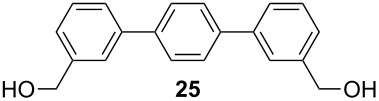	75
15	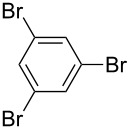	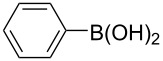	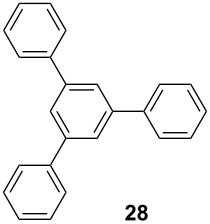	91
16	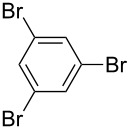	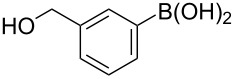	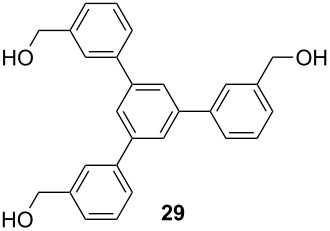	80

^a^Reaction conditions: aryl halide (1 mmol), arylboronic acid (3.5 mmol), base (2 mmol), 4 mL of 1:1 DMF/H_2_O and Pd/NiFe_2_O_4_ (1 mol %) at 90 °C for 2 h. ^b^After purification by flash silica gel chromatography.

The feasibility of recycling the Pd/NiFe_2_O_4_ was examined (Supporting Information File 1). The recycling experiment was performed on the model reaction (iodobenzene with phenylboronic acid). After completion of the reaction the catalyst was removed by applying an external magnet and washed with dichloromethane and water several times. The oven dried (100 °C) catalyst was then used for the next run. The Pd/NiFe_2_O_4_ catalyst exhibited only a marginal change in the catalytic activity and required a longer reaction time to achieve the similar conversion after the fourth cycle.

In the literature, several mechanisms have been considered for the interaction of the Pd catalyst with the substrate and intermediates [62]. It has become more and more accepted that the Pd in solution as a colloid or complexed is the true catalytically active species for the heterogeneous catalyzed reaction [28,62]. We believe that, the nickel ferrite activates the Pd surface which helps better desorption and leads to faster formation of colloids or complexes in the solution, and that the palladium is redeposited onto the support or Pd clusters on completion of the reaction. Thus, Pd/NiFe_2_O_4_ acts like a reservoir for the dissolved Pd species, in a different phase from that of reactants and products. To confirm this assumption, we performed the model reaction between iodobenzene and phenylboronic acid. After the reaction was complete, Pd/NiFe_2_O_4_ catalyst was removed from the hot reaction mixture by applying an external magnet and the clear solution filtered to remove any dispersed particles. To the clear filtrate, the appropriate amounts of new substrates, i.e., 4-iodotoluene, phenylboronic acid and base was added. The composition of the reaction mixture was analyzed by GC, the amount of 4-iodotoluene was set to 100% and the reaction was performed under the identical reaction conditions. It was observed that 54% of 4-iodotoluene was converted to the corresponding biaryl after 60 min and the Pd content detected in the filtrate was 2.3 ppm based on AAS analysis. In another set of experiment, we heated the freshly prepared Pd/NiFe_2_O_4_ catalyst at 90 °C in 1:1 DMF/H_2_O solvent for 30 min. After separating the hot solid catalyst, the clear filtrate was mixed with the appropriate amounts of iodobenzene, phenylboronic acid and Na_2_CO_3_ and the Suzuki reaction carried out at 90 °C. After heating for 60 min, only 29% iodobenzene was converted to biphenyl and 1.1 ppm of Pd was detected in the solution. This suggested that the oxidative addition of the iodobenzene to the surface Pd(0) converted Pd(0) into Pd(II) and resulted in the leaching of a certain amount of palladium particles into the solution. The experimental results showed that the leaching of palladium was required for this sustaining catalytic activity and nickel ferrite supported palladium provides the source of palladium in the process.

## Conclusion

In summary, we have extensively studied the Pd/NiFe_2_O_4_ catalyst which showed high catalytic activity in the Suzuki reaction in aqueous DMF in short reaction times. The salient features of the present methodology are that it is ligand free, heterogeneous, uses water as a co-solvent and requires no additives. Moreover, the method can be extended to the synthesis of various polyaryls in a single step with high yield and selectivity under aerobic conditions in aqueous solvents. The Pd/NiFe_2_O_4_ catalyst can be easily removed from the reaction mixture by applying an external magnetic field and reused several times without significant loss of catalytic activity. The studies reveal that the palladium leaching into the solution provides the catalytic site.

## Experimental

### General

All experiments and manipulations were performed in an ambient atmosphere. All glassware was washed with acid then base and oven dried before use. The catalytic reactions were carried out in round bottom flasks equipped with a reflux condenser. The [Pd(NH_3_)_4_](OAc)_2_, reactants and solvents were obtained from Sigma–Aldrich/Merck or Spectrochem and used as received without further purification or drying. The progress of reaction was monitored by GC (HP-5890, series II, equipped with HP-5 capillary column and a FID detector, using N_2_ as the carrier gas). The products were isolated by extraction with ethyl acetate and purified by silica gel (100–200) column chromatography. The isolated products were characterized by GC–MS (Shimadzu QP-5050, DB-5 column and a TCD detector with He as the carrier gas), FTIR (8400 Shimadzu), and NMR spectroscopy (Varian 300 MHz spectrometer in CDCl_3_ or DMSO-*d*_6_ with TMS as an internal standard). The Pd content was measured by Atomic Absorption Spectrophotometer (AAS), Model no: Chemito AA-201.

#### General procedure for the synthesis of NiFe_2_O_4_

The metal oxalate precursor was prepared by adding 32 mL of a N_2_ purged solution of NiSO_4_·6H_2_O (3.0 g, 0.0114 mol) and FeSO_4_·7H_2_O (6.82 g, 0.0228 mol) to the 80 mL of N_2_ purged (COONH_4_)_2_·H_2_O (3.65 g, 0.0228 mol) with vigorous stirring. The resulting yellowish green suspension was cooled to 0 °C and the supernatant liquid decanted before filtration. The precipitate was washed with ~250 mL distilled water and dried in an oven at 110 °C for 1 h. The nickel ferrite was obtained by heating the above precipitate in air for 4 h at 550 °C in a silica crucible in a pre-heated muffle furnace.

#### Typical procedure for the palladium deposition onto NiFe_2_O_4_

To a suspension of NiFe_2_O_4_ (1.0 g) in absolute ethanol (100 mL), [Pd(NH_3_)_4_](OAc)_2_ (0.010 g) was added. The brownish slurry was stirred for 6 h at room temperature. The solvent was then evaporated under reduced pressure on rotary evaporator. The Pd-impregnated solid was dried in oven at 100 °C for 3 h, then reduced under a continuous stream of hydrogen gas (20 mL/min) at 200 °C for 4 h to give a black powder (BET surface area = 29 m^2^/g).

#### General procedure for Suzuki reaction catalyzed by Pd/NiFe_2_O_4_

To a 25 mL two neck round bottom flask attached with a reflux condenser, were added the aryl halide (1 mmol), boronic acid (1.25 mmol), Na_2_CO_3_ (2 mmol), and Pd/NiFe_2_O_4_ (0.1 mol %) in 4 mL DMF/H_2_O (1:1), and the reaction mixture heated at the appropriate temperature and duration. The reaction was monitored by gas chromatography. After the reaction was complete, the mixture was extracted with ethyl acetate three times, the combined organic extracts dried over anhydrous Na_2_SO_4_ and the solvent evaporated at reduced pressure. The crude products were then purified by column chromatography [hexane or hexane/ethyl acetate (9:1)] and analyzed by GC, GC–MS, IR, and NMR.

## Supporting Information

Scanning electron microscope image, X-ray photoemission spectrum, X-ray powder diffraction pattern, catalytic activity of different loading of palladium over NiFe_2_O_4_, catalyst recycling studies and ^1^H and ^13^C NMR for the products **1**–**29**.

File 1 Characterization data of the catalyst and of the products **1**–**29**.

## References

[1] Suzuki A, Diederich F, Stang P J (1998). Cross-coupling Reactions of Organoboron Compounds with Organic Halides. Metal-Catalyzed Cross-Coupling Reactions.

[2] Tsuji J (2004). Palladium Reagents and Catalysts: New Perspectives for the 21st Century.

[3] Negishi E (2002). Handbook of Organopalladium Chemistry for Organic Synthesis.

[4] Nicolaou K C, Bulger P G, Sarlah D (2005). Angew Chem, Int Ed.

[5] Miyaura N, Suzuki A (1995). Chem Rev.

[6] Stanforth S P (1998). Tetrahedron.

[7] Lloyd-Williams P, Giralt E (2001). Chem Soc Rev.

[8] Hassan J, Sévignon M, Gozzi C, Schulz E, Lemaire M (2002). Chem Rev.

[9] Pu L (1998). Chem Rev.

[10] Persichini P J (2003). Curr Org Chem.

[11] Corbet J-P, Mignani G (2006). Chem Rev.

[12] Alonso F, Beletskaya I P, Yus M (2008). Tetrahedron.

[13] Phan N T S, Van Der Sluys M, Jones C W (2006). Adv Synth Catal.

[14] Mino T, Shirae Y, Sakamoto M, Fujita T (2005). J Org Chem.

[15] Cui X, Zhou Y, Wang N, Liu L, Guo Q-X (2007). Tetrahedron Lett.

[16] Borhade S R, Waghmode S B (2008). Tetrahedron Lett.

[17] Li S, Lin Y, Cao J, Zhang S (2007). J Org Chem.

[18] So C M, Lau C P, Kwong F Y (2007). Org Lett.

[19] Marion N, Navarro O, Mei J, Stevens E D, Scott N M, Nolan S P (2006). J Am Chem Soc.

[20] Hartley F R (1985). Supported Metal Complexes: A New Generation of Catalysts.

[21] Garrett C E, Prasad K (2004). Adv Synth Catal.

[22] Yin L, Liebscher J (2007). Chem Rev.

[23] Taylor R H, Felpin F-X (2007). Org Lett.

[24] Felpin F-X, Ayad T, Mitra S (2006). Eur J Org Chem.

[25] Zhang Z, Wang Z (2006). J Org Chem.

[26] Yang Q, Ma S, Li J, Xiao F, Xiong H (2006). Chem Commun.

[27] Sayah R, Glegoła K, Framery E, Dufaud V (2007). Adv Synth Catal.

[28] Wong H, Pink C J, Ferreira F C, Livingston A G (2006). Green Chem.

[29] Köhler K, Heidenreich R G, Soomro S S, Pröckl S S (2008). Adv Synth Catal.

[30] Jana S, Haldar S, Koner S (2009). Tetrahedron Lett.

[31] Fan G, Zou B, Cheng S, Zheng L (2010). J Ind Eng Chem.

[32] Scheuermann G M, Rumi L, Steurer P, Bannwarth W, Mülhaupt R M (2009). J Am Chem Soc.

[33] Li C-J, Chan T-H (1997). Organic Reactions in Aqueous Media.

[34] Grieco P A (1997). Organic Synthesis in Water.

[35] Sheldon R A (2005). Green Chem.

[36] Beletskaya I P, Cheprakov A V, Negishi E (2002). Handbook of Organopalladium Chemistry for Organic Synthesis.

[37] Shaughnessy K H (2006). Eur J Org Chem.

[38] Shaughnessy K H, Booth R S (2001). Org Lett.

[39] Gelpke A E S, Veerman J J N, Goedheijt M S, Kamer P C J, van Leeuwen P W N M, Hiemstra H (1999). Tetrahedron.

[40] Baruwati B, Guin D, Manorama S V (2007). Org Lett.

[41] Borhade S R, Waghmode S B (2008). Indian J Chem, Sect B.

[42] McGarvey G B, Owen D G (1998). J Mater Sci.

[43] Palczewska W (1975). Adv Catal.

[44] Liu J-K (2006). Chem Rev.

[45] Zhang C, Ondeyka J G, Herath K B, Guan Z, Collado J, Pelaez F, Leavitt P S, Gurnett A, Nare B, Liberator P (2006). J Nat Prod.

[46] Roberti M, Pizzirani D, Recanatini M, Simoni D, Grimaudo S, Di Cristina A, Abbadessa V, Gebbia N, Tolomeo M (2006). J Med Chem.

[47] Lee I-K, Jung J-Y, Kim Y-S, Rhee M H, Yun B-S (2009). Bioorg Med Chem.

[48] Ichimura K (2000). Chem Rev.

[49] Watson M D, Fechtenkötter A, Müllen K (2001). Chem Rev.

[50] Gary G W, Winsor P A (1974). Liquid Crystals and Plastic Crystals 1.

[51] Hall N (2003). Chem Commun.

[52] Pron A, Rannou P (2002). Prog Polym Sci.

[53] Rottländer M, Knochel P (1998). J Org Chem.

[54] Li S, Wei B, Low P M N, Lee H K, Hor T S A, Xue F, Mak T C W (1997). J Chem Soc, Dalton Trans.

[55] Minato A, Tamao K, Hayashi T, Suzuki K, Kumada M (1980). Tetrahedron Lett.

[56] Nakada M, Miura C, Nishiyama H, Higashi F, Mori T, Hirota M, Ishii T (1989). Bull Chem Soc Jpn.

[57] Sinclair D J, Sherburn M S (2005). J Org Chem.

[58] Liu L, Zhang Y, Xin B (2006). J Org Chem.

[59] Miguez J M A, Adrio L A, Sousa-Pedrares A, Vila J M, Hii K K (2007). J Org Chem.

[60] Sharif M, Zeeshan M, Reimann S, Villinger A, Langer P (2010). Tetrahedron Lett.

[61] Paul S, Clark J H (2003). Green Chem.

[62] Wu K M, Huang C A, Peng K F, Chen C T (2005). Tetrahedron.

