# Supplementary material for: Studies on Pd/NiFe2O4 catalyzed ligand-free Suzuki reaction in aqueous phase: synthesis of biaryls, terphenyls and polyaryls
Source: Beilstein J Org Chem. 2011 Mar 15;7:310–9. doi: 10.3762/bjoc.7.41 (PMC3063056; doi:10.3762/bjoc.7.41)
Supplement: File 1 — Characterization data of the catalyst and of the products 1–29. [file Beilstein_J_Org_Chem-07-310-s001.pdf]

Supporting Information

for

Studies on Pd/$NiFe_2O_4$ catalyzed ligand-free Suzuki reaction in aqueous phase: synthesis of biaryls, terphenyls and polyaryls

Sanjay R. Borhade and Suresh B. Waghmode*[§]

Address: Department of Chemistry, University of Pune, Ganeshkhind, Pune-411007, India

Email: Suresh B. Waghmode - suresh@chem.unipune.ac.in

[§]Phone +91 20 2560 1225 (extension) 545 and 585; Fax +91 20 2569 1728

*Corresponding author

Characterization data of the catalyst and of the products 1–29

Content **Page No**

1. Figure 1: Scanning electron microscope image for the Pd/$NiFe_2O_4$......................................2

2. Figure 2: The X-ray photoemission spectra of Pd/$NiFe_2O_4$...3

3. Figure 3: Effect of various concentrations on the Suzuki coupling reaction..........................4

4. Figure 4: X-ray diffraction pattern for the fresh and spent Pd/$NiFe_2O_4$ catalyst....................5

5. Table 1: Recycling of Pd/$NiFe_2O_4$...6

6. Characterization data of the products **1–29**...7

7. References..16

1. Figure 1: Scanning electron microscope image for the Pd/$NiFe_2O_4$.

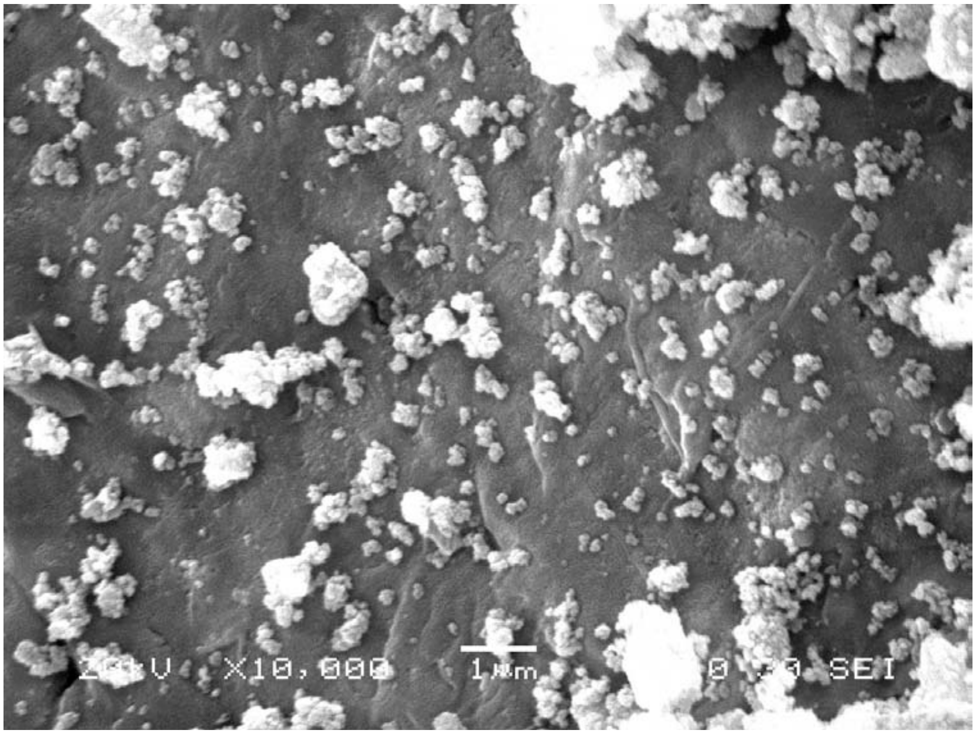

2. Figure 2: The X-ray photoemission spectrum of Pd/$NiFe_2O_4$.

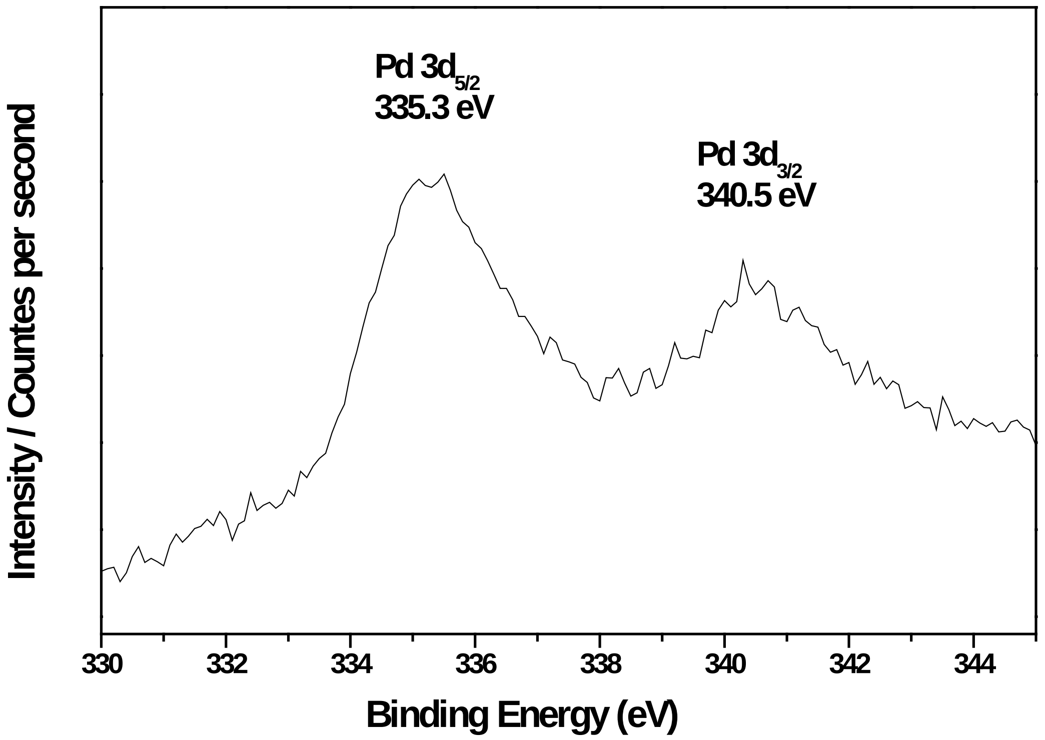

3. Figure 3: Effect of various concentrations on the Suzuki coupling reaction[a].

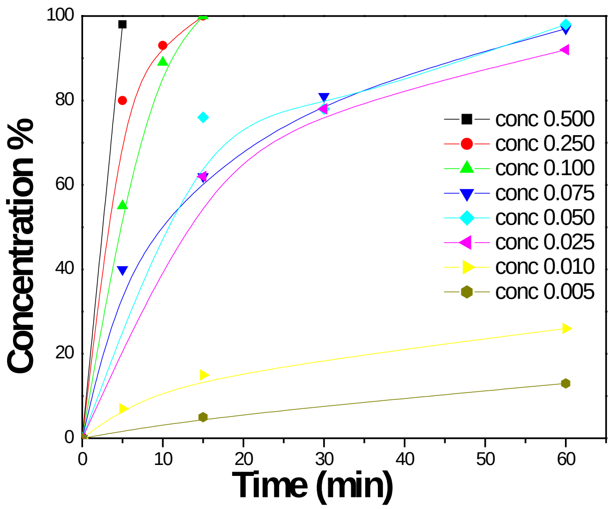

[a]Reaction conditions: iodobenzene (1 mmol), phenylboronic acid (1.2 mmol), base (2 mmol), 4 mL of 1:1 H_2O/DMF, at 90 °C and at different Pd concentrations.

4. Figure 4: X-ray diffraction pattern for the fresh and spent Pd/$NiFe_2O_4$ catalyst.

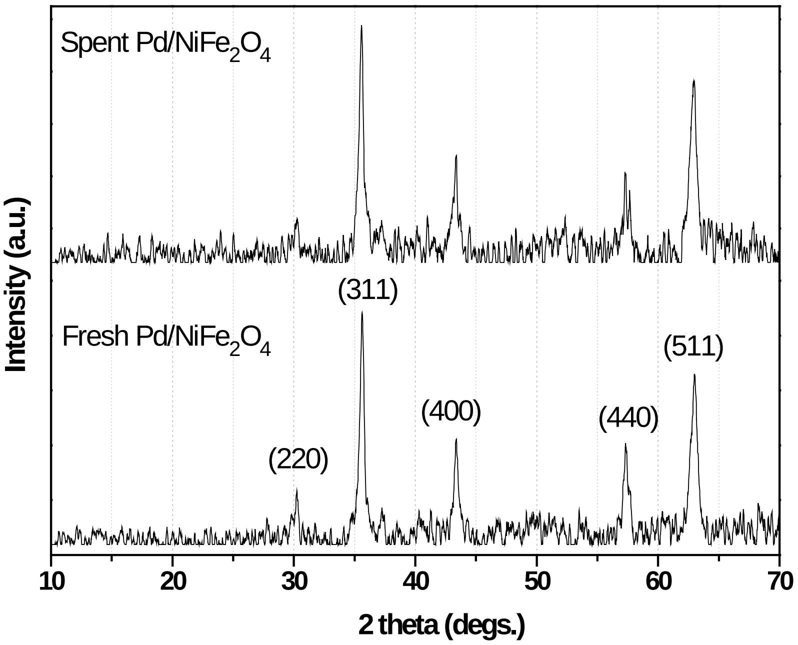

5. Table 1: Recycling of $Pd/NiFe_2O_4$ for the Suzuki reaction of iodobenzene with phenylboronic acid[a]

Entry	Catalyst	Time (min)	% Conversion[b]
1	first cycle	10	100
2	second cycle	20	100
3	third cycle	20	98
4	fourth cycle	20	91
5	fifth cycle	30	95

[a]Reaction conditions: iodobenzene (1 mmol), phenylboronic acid (1.2 mmol), Na_2CO_3 (2 mmol), 4 mL of 1:1 H_2O/DMF and $Pd/NiFe_2O_4$ (0.1 mol %) at 90 °C.

[b]Conversions were determined by GC (Δrel = ±5 %).

6. Characterization data for the products

Biphenyl [**1**, 92-52-4, Ref. 1]

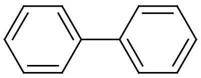

^{1}H NMR (300 MHz, $CDCl_3$, TMS): δ 7.27–7.31 (m, 2H), 7.40 (t, 4H, *J* = 7.1 Hz), 7.54 (d, 4H, *J* = 7.1 Hz), ^{13}C NMR (75 MHz, $CDCl_3$, TMS): δ 127.0, 127.1, 128.6, 141.0. FTIR (KBr, cm^{-1}): 1429, 1479, 3036. MS (EI): *m/z* 154 (M^+).

3-(Hydroxymethyl)biphenyl [**2**, 69605-90-6, Ref. 2]

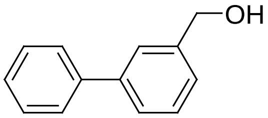

^{1}H NMR (300 MHz, $CDCl_3$, TMS): δ 2.29 (s, 1H), 4.66 (s, 2H), 7.25–7.55 (m, 9H). ^{13}C NMR (75 MHz, $CDCl_3$, TMS): δ 65.1, 125.6, 125.7, 126.2, 127.0, 127.2, 128.6, 128.8, 140.7, 141.1, 141.3. FTIR (KBr, cm^{-1}): 1186, 1454, 1477, 3313-3381. MS (EI): *m/z* 184 (M^+).

3-Carbethoxybiphenyl [**3**, 19926-50-2, Ref. 3]

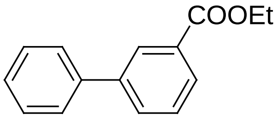

^{1}H NMR (300 MHz, $CDCl_3$, TMS): δ 1.43 (t, 3H, *J* = 7.1 Hz), 4.41 (q, 2H, *J* = 7.1 Hz), 7.38–7.47 (m, 4H), 7.61 (d, 2H, 7.7 Hz), 7.77 (d, 1H, *J* = 7.7 Hz), 8.01 (d, 1H, *J* = 7.7 Hz) 8.27 (s, 1H). ^{13}C NMR (75 MHz, $CDCl_3$, TMS): δ 14.4, 61.0, 127.0, 127.6, 128.1, 128.2, 128.7, 128.7, 130.9, 131.3, 140.0, 141.2, 166.3. FTIR (KBr, cm^{-1}): 2982, 1718, 1593, 1301, 1242. MS (EI): *m/z* 226 (M^+).

3-Aminobiphenyl [**4**, 2243-47-2, Ref. 4]

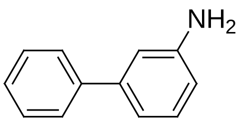

^{1}H NMR (300 MHz, $CDCl_3$+2 drops of DMSO-d_6, TMS): δ 3.78 (bs, 2H), 6.67 (d, 1H, *J* = 7.1 Hz), 6.92 (d, 1H, *J* = 6.6 Hz), 6.93 (s, 1H), 7.17 (t, 1H, *J* = 7.9 Hz), 7.29 (d, 1H, *J* = 7.1 Hz) 7.38 (t, 2H, *J* = 7.7 Hz), 7.52 (d, 2H, *J* = 7.1 Hz). ^{13}C NMR

(75 MHz, $CDCl_3$+2 drops of DMSO-d_6, TMS): δ 113.0, 113.6, 116.2, 125.8, 126.2, 127.6, 128.6, 140.1, 140.9, 145.4. FTIR (neat, cm^{-1}): 3446, 3369, 3054, 1618, 1575, 1481, 1317, 1226. MS (EI): *m/z* 169 (M^+).

2-Amino-5-phenylpyrimidine [**5**, 31408-23-8, Ref. 5]

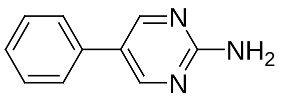

^{1}H NMR (300 MHz, $CDCl_3$, TMS): δ 5.60 (bs, 2H), 7.36–7.47 (m, 5H), 8.52 (s, 2H). ^{13}C NMR (75 MHz, $CDCl_3$, TMS): δ 124.6, 125.8, 127.41, 129.0, 135.0, 156.2, 162.0. FTIR (KBr, cm^{-1}): 3178, 3323, 1600, 1518, 1136. MS (EI): *m/z* 171 (M^+).

3-Methoxy-5-phenylpyridine [**6**, 53698-52-5, Ref. 6]

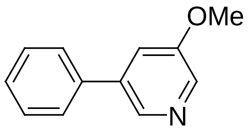

^{1}H NMR (300 MHz, $CDCl_3$, TMS): δ 3.90 (s, 3H), 7.35–7.56 (m, 6H), 8.27 (s, 1H), 8.44 (s, 1H). ^{13}C NMR (75 MHz, $CDCl_3$, TMS): δ 55.6, 119.1, 127.1, 128.1, 128.9, 135.6, 137.4, 140.3, 155.5. FTIR (KBr, cm^{-1}): 1413, 1498, 1587, 2929, 3057. MS (EI): *m/z* 185 (M^+).

4-Hydroxybiphenyl [**7**, 92-69-3, Ref. 7]

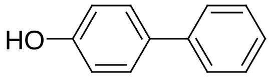

^{1}H NMR (300 MHz, $CDCl_3$, TMS): δ 4.51 (br s, 1H), 6.89 (d, 2H, J = 8.5 Hz), 7.24–7.55 (m, 7H). ^{13}C NMR (75 MHz, $CDCl_3$, TMS): δ 115.5, 126.6, 128.2, 128.6, 133.7, 140.6, 155.0. FTIR (KBr, cm^{-1}): 1487, 1597, 3037, 3416. MS (EI): *m/z* 170 (M^+).

2-(Trifluromethyl)biphenyl [**8**, 362-59-4, Ref. 8]

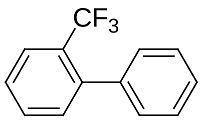

^{1}H NMR (300 MHz, $CDCl_3$, TMS): δ 7.27–7.49 (m, 8H), 7.70 (d, 1H, *J* = 7.7 Hz). ^{13}C NMR (75 MHz, $CDCl_3$, TMS): δ 122.7, 126.3 (q), 126.4, 127.5, 127.6, 127.9, 128.0, 128.6, 129.3, 131.6, 132.4, 140.2, 141.7. FTIR (KBr, cm^{-1}): 1315, 1483, 1600, 2926. MS (EI): *m/z* 222 (M^+).

2-Hydroxybiphenyl [**9**, 90-43-7, Ref. 9]

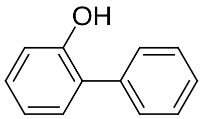

^{1}H NMR (300 MHz, $CDCl_3$, TMS): δ 5.23 (bs, 1H), 6.94 (t, 2H), 7.20 (t, 2H), 7.33–7.41 (m, 5H). ^{13}C NMR (75 MHz, $CDCl_3$, TMS): δ 115.7, 120.7, 127.71, 128.0, 129.0, 129.1, 130.1, 136.9, 152.8. FTIR (KBr, cm^{-1}): 1101, 1327, 1481, 1585, 3028, 3535–3558. MS (EI): *m/z* 170 (M^+).

2-Aminobiphenyl [**10**, 90-41-5, Ref. 10]

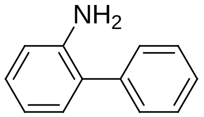

^{1}H NMR (300 MHz, $CDCl_3$, TMS): δ 3.74 (bs, 2H), 6.81(d, 1H, *J* = 7.7 Hz), 6.88 (t, 1H), 7.21(m, 2H), 7.41 (m, 1H), 7.49 (m, 4H), ^{13}C NMR (75 MHz, $CDCl_3$, TMS): δ 115.5, 118.5, 127.0, 127.5, 128.3, 128.6, 128.9, 130.3, 139.3, 143.2. FTIR (KBr, cm^{-1}): 1292, 1481, 1612, 3022, 3387, 3479. MS (EI): *m/z* 169 (M^+).

2-Methoxybiphenyl [**11**, 86-26-0, Ref. 11]

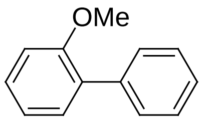

^{1}H NMR (300 MHz, $CDCl_3$, TMS): δ 3.77 (s, 3H), 6.92–6.98 (m, 2H), 7.25–7.30 (m, 3H), 7.34 (t, 2H), 7.51 (d, 2H, *J* = 6.8 Hz). ^{13}C NMR (75 MHz, $CDCl_3$, TMS): δ 55.5, 111.0, 120.7, 126.8, 127.8, 128.5, 129.4, 130.5, 130.7, 138.3, 156.2. FTIR (KBr, cm^{-1}): 1247, 1467, 1599. MS (EI): *m/z* 184 (M^+).

4-Methoxy-[3-(hydroxymethyl)phenyl]benzene [**12**, 20854-56-2, Ref. 12]

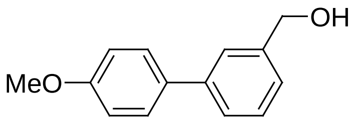

^{1}H NMR (300 MHz, $CDCl_3$, TMS): δ 1.79 (s, 1H), 3.83 (s, 3H), 4.72 (s, 2H), 6.93 (d, 2H, 8.8 Hz), 7.26 (d, 1H, *J* = 7.4 Hz), 7.38 (t, 1H, *J* = 7.7 Hz), 7.44–7.54 (m, 4H). ^{13}C NMR (75 MHz, $CDCl_3$, TMS): δ 55.3, 65.4, 114.1, 125.2, 125.2, 125.9, 128.0, 128.8, 133.3, 141.0, 141.2, 159.0. FTIR (KBr, cm^{-1}): 3254, 1604, 1516, 1251, 1031. MS (EI): *m/z* 214 (M^+).

4-Methoxybiphenyl [**13**, 613-37-6, Ref. 13]

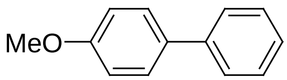

^{1}H NMR (300 MHz, $CDCl_3$, TMS): δ 3.82 (s, 3H), 6.93 (d, 2H, *J* = 8.5 Hz), 7.27 (t, 1H), 7.38 (t, 2H), 7.51 (t, 4H). ^{13}C NMR (75 MHz, $CDCl_3$, TMS): δ 55.3, 114.1, 126.6, 128.0, 128.6, 133.6, 140.6, 158.9. FTIR (KBr, cm^{-1}): 1037, 1247, 1485, 1604. MS (EI): *m/z* 184 (M^+).

4-Methylbiphenyl [**14**, 644-08-06, Ref. 13]

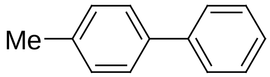

^{1}H NMR (300 MHz, $CDCl_3$, TMS): δ 2.37 (s, 3H), 7.20 (d, 2H, *J* = 7.7 Hz), 7.28 (t, 1H), 7.38 (t, 2H), 7.45 (d, 2H, *J* = 7.9 Hz), 7.54 (d, 2H, *J* = 7.4 Hz). ^{13}C NMR (75 MHz, $CDCl_3$, TMS): δ 21.2, 126.9, 128.6, 129.3, 136.8, 138.2, 141.0. FTIR (KBr, cm^{-1}): 1485, 3030. MS (EI): *m/z* 168 (M^+).

4-Methyl-[3-(hydroxymethyl)phenyl]benzene [**15**, 89951-79-1, Ref. 14]

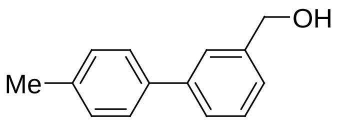

^{1}H NMR (300 MHz, $CDCl_3$, TMS): δ 1.95 (s, 1H), 2.37 (s, 3H), 4.70 (s, 2H), 7.20 (d, 2H, 7.7 Hz), 7.27 (d, 1H, *J* = 7.7 Hz), 7.37 (t, 1H, *J* = 7.4 Hz), 7.45 (d, 3H, *J* = 7.7 Hz) 7.54 (s, 1H). ^{13}C NMR (75 MHz, $CDCl_3$, TMS): δ 21.2,

65.3, 125.5, 126.1, 126.8, 128.8, 129.4, 137.0, 137.9, 141.1, 141.3. FTIR (KBr, cm^{-1}): 3375, 3302, 2916, 1608, 1485, 1342, 1190. MS (EI): *m/z* 198 (M^+).

4-Nitro-[3-(hydroxymethyl)phenyl]benzene [**16**, 62038-00-0, Ref. 6]

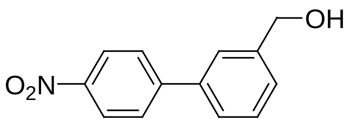

^1H NMR (300 MHz, $CDCl_3$, TMS): δ 2.05 (s, 1H), 4.78 (s, 2H), 7.42–7.51 (m, 4H), 7.61 (s, 1H), 7.70 (d, 2H, *J* = 8.5 Hz), 8.25 (d, 2H, *J* = 8.5 Hz). ^{13}C NMR (75 MHz, $CDCl_3$, TMS): δ 64.9, 123.9, 125.7, 126.4, 127.2, 127.7, 129.2, 138.8, 141.7, 146.8, 147.2. FTIR (KBr, cm^{-1}): 1035, 1346, 1516, 1597, 2929, 3282–3373. MS (EI): m/z 229 (M^+).

4-Nitrobiphenyl [**17**, 92-93-3, Ref. 13]

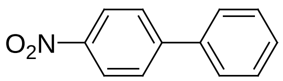

^1H NMR (300 MHz, $CDCl_3$, TMS): δ 7.42-7.50 (m, 3H), 7.60 (d, 2H, *J* = 6.8 Hz), 7.71 (d, 2H, *J* = 8.8 Hz), 8.27 (d, 2H, 8.8 Hz). ^{13}C NMR (75 MHz, $CDCl_3$, TMS): δ 124.0, 127.2, 127.7, 128.8, 129.0, 138.6, 146.9, 147.4. FTIR (KBr, cm^{-1}): 1109, 1348, 1512, 1595, 3076. MS (EI): *m/z* 199 (M^+).

4-Chloro-[3-(hydroxymethyl)phenyl]benzene [**18**, 773872-39-2, Ref. 6]

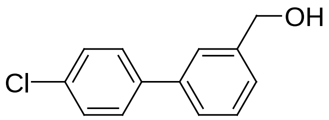

^1H NMR (300 MHz, $CDCl_3$, TMS): δ 1.93 (s, 1H), 4.72 (s, 2H), 7.35 (m, 4H), 7.47 (m, 4H). ^{13}C NMR (75 MHz, $CDCl_3$, TMS): δ 65.2, 125.4, 126.0, 126.1, 128.2, 128.8, 129.0, 133.3, 139.2, 140.1, 141.3. FTIR (KBr, cm^{-1}): 3348, 1475, 1433, 1338, 1188. MS (EI): *m/z* 218 (M^+).

4-Chlorobiphenyl [**19**, 2051-62-9, Ref. 15]

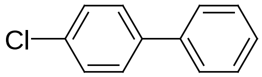

^{1}H NMR (300 MHz, $CDCl_3$, TMS): δ 7.28–7.51 (m, 9H), ^{13}C NMR (75 MHz, $CDCl_3$, TMS): δ 126.8, 127.4, 128.2, 128.7, 133.2, 139.4, 139.8. FTIR (KBr, cm^{-1}): 1099, 1479, 1591, 3063. MS (EI): *m/z* 188 (M^+).

4-Phenylbenzaldehyde [**20**, 3218-36-8, Ref. 1]

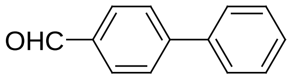

^{1}H NMR (300 MHz, $CDCl_3$, TMS): δ 7.36–7.47 (m, 3H), 7.59 (d, 2H, *J* = 7.7 Hz), 7.71 (d, 2H, *J* = 7.9 Hz), 7.91 (d, 2H, 8.2 Hz), 10.01 (s, 1H), ^{13}C NMR (75 MHz, $CDCl_3$, TMS): δ 127.2, 127.5, 128.3, 128.8, 130.1, 135.0, 139.5, 147.0, 191.7. FTIR (KBr, cm^{-1}): 1217, 1481, 1602, 1689, 2746, 2835, 3026. MS (EI): *m/z* 182 (M^+).

4-Acetylbiphenyl [**21**, 92-91-1, Ref. 15]

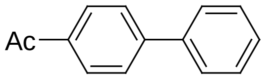

^{1}H NMR (300 MHz, $CDCl_3$, TMS): δ 2.63 (s, 3H), 7.37–7.47 (m, 3H), 7.59 (d, 2H, *J* = 7.1 Hz), 7.66 (d, 2H, *J* = 7.9 Hz), 8.01 (d, 2H, *J* = 8.25 Hz). ^{13}C NMR (75 MHz, $CDCl_3$, TMS): δ 26.8, 127.1, 128.1, 128.8, 135.7, 139.7, 145.6, 198.8. FTIR (KBr, cm^{-1}): 2999, 1678, 1600, 1558, 1263. MS (EI): *m/z* 196 (M^+).

1,2-Diphenylbenzene [**22**, 84-15-1, Ref. 16]

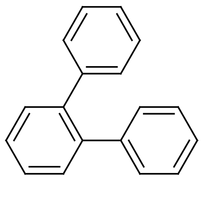

^{1}H NMR (300 MHz, $CDCl_3$, TMS): δ 7.21–7.38 (m, 10H), 7.46 (s, 4H). ^{13}C NMR (75 MHz, $CDCl_3$, TMS): δ 126.3, 127.0, 127.14, 127.3, 127.7, 128.6, 129.8, 130.5, 140.4, 141.4. FTIR (KBr, cm^{-1}): 1265, 1421, 1618, 2924. MS (EI): *m/z* 230 (M^+).

1,2-bis[(3-hydroxymethyl)phenyl]benzene [**23**, New compound]

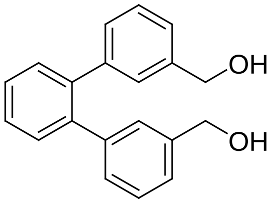

^{1}H NMR (300 MHz, $CDCl_3$, TMS): δ 3.24 (s, 2H), 4.40 (s, 4H), 7.01–7.18 (m, 8H), 7.39–7.43 (m, 4H), ^{13}C NMR (75 MHz, $CDCl_3$, TMS): δ 64.7, 125.2, 127.4, 127.9, 128.8, 130.1, 140.1, 141.3. FTIR (KBr, cm^{-1}): 1045, 1406, 1460, 1624, 3416. MS (EI): *m/z* 290 (M^+). Anal. Calcd for $C_{20}H_{18}O_2$: C, 82.73; H, 6.25; O, 11.02. Found: C, 82.71; H, 6.28.

1,4-Diphenylbenzene [**24**, 92-94-4, Ref. 17]

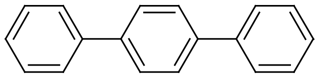

^{1}H NMR (300 MHz, $CDCl_3$, TMS): δ 7.36 (t, 2H, *J* = 7.1 Hz), 7.45 (t, 4H, *J* = 7.5 Hz), 7.62 (d, 4H, *J* = 7.1 Hz), 7.65 (s, 4H). ^{13}C NMR (75 MHz, $CDCl_3$, TMS): δ 126.9, 127.2, 127.4, 128.7, 140.0, 140.5. FTIR (KBr, cm^{-1}): 3034, 1604, 1479. MS (EI): *m/z* 230 (M^+).

1,4-Bis[3-(hydroxymethyl)phenyl]benzene [**25**, New compound]

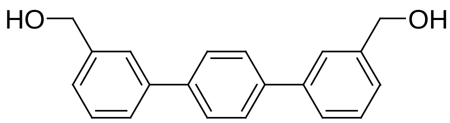

^{1}H NMR (300 MHz, $CDCl_3$, TMS): δ 4.57 (d, 4H), 5.28 (t, 2H), 7.32 (d, *J* = 7.42 Hz, 2H), 7.43 (t, *J* = 7.70 Hz, 2H), 7.58 (d, *J* = 7.70 Hz, 2H), 7.67 (s, 2H), 7.76 (s, 4H). ^{13}C NMR (75 MHz, $CDCl_3$, TMS): δ 62.9, 124.6, 125.6, 127.1, 128.7, 139.2, 139.3, 143.3. FTIR (KBr, cm^{-1}): 1008, 1201, 1433, 1481, 1604, 2929, 3417-3444. MS (DI): *m/z* 290 (M^+). Anal. Calcd for $C_{20}H_{18}O_2$: C, 82.73; H, 6.25; O, 11.02. Found: C, 82.52; H, 6.10.

1,3-Diphenylbenzene [**26**, 92-06-8, Ref. 17]

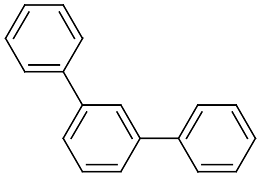

^{1}H NMR (300 MHz, $CDCl_3$, TMS): δ 7.33–7.63 (m, 13H), 7.79 (s, 3H), ^{13}C NMR (75 MHz, $CDCl_3$, TMS): δ 126.0, 127.1, 127.3, 128.7, 129.0, 141.02, 141.6. FTIR (KBr, cm^{-1}):3057, 3032, 1597, 1568, 1496, 1402. MS (EI): *m/z* 230 (M^+).

1,3-Bis[3-(hydroxymethyl)phenyl]benzene [**27**, New compound]

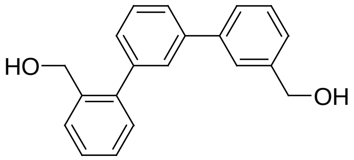

^{1}H NMR (300 MHz, $CDCl_3$, TMS): δ 3.21 (s, 2H), 4.58 (s, 4H), 7.19 (d, *J* = 7.1 Hz, 2H), 7.26–7.43 (m, 7H), 7.50 (s, 2H), 7.67 (s, 1H). ^{13}C NMR (75 MHz, $CDCl_3$, TMS): δ 64.8, 125.4, 125.5, 125.7, 125.8, 125.9, 126.0, 126.1, 128.7, 129.0, 141.0, 141.2, 141.2. FTIR (KBr, cm^{-1}): 1028, 1265, 1417, 1602, 2926, 3412. MS (DI): *m/z* 290 (M^+). Anal. Calcd for $C_{20}H_{18}O_2$: C, 82.73; H, 6.25; O, 11.02. Found: C, 82.64; H, 6.24.

1,3,5-Triphenylbenzene [**28**, 612-71-5, Ref. 16]

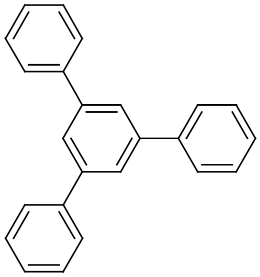

^{1}H NMR (300 MHz, $CDCl_3$, TMS): δ 7.37 (t, 3H, *J* = 7.1 Hz), 7.46 (t, 5H, *J* = 7.7 Hz), 7.68 (d, 5H, *J* = 7.45 Hz), 7.77 (s, 3H). ^{13}C NMR (75 MHz, $CDCl_3$, TMS): δ 125.1, 127.2, 127.4, 128.4, 141.0, 142.2. FTIR (KBr, cm^{-1}): 3057, 3033, 1593, 1494, 1410. MS (EI): *m/z* 306 (M^+).

1,3,5-Tris[3-(hydroxymethyl)phenyl]benzene [**29**, New compound]

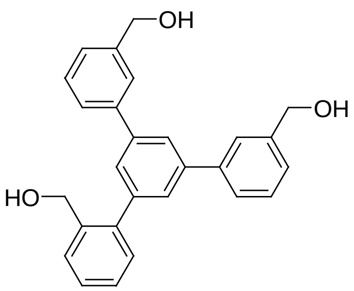

^{1}H NMR (300 MHz, DMSO-d_6, TMS): δ 4.61 (d, 6H, *J* = 5.7 Hz), 5.30 (t, 3H, *J* = 5.7 Hz), 7.36 (d, 3H, *J* = 7.4 Hz), 7.46 (t, 3H, *J* = 7.4 Hz), 7.70 (d, 3H, *J* = 7.4 Hz) 7.76 (s, 3H), 7.85 (s, 3H). ^{13}C NMR (75 MHz, DMSO-d_6, TMS): δ 62.9, 124.1, 125.0, 125.3, 125.8, 128.6, 139.7, 141.6, 143.1. FTIR (KBr, cm^{-1}): 3281, 2870, 1595, 1585, 1400. MS (DI): m/z 396 (M^+). Anal. Calcd for $C_{27}H_{24}O_3$: C, 81.79; H, 6.10; O, 12.11. Found: C, 81.77; H, 6.18.

7. **References**

[1] J. Mao, J. Guo, F. Fang, S. J. Ji, *Tetrahedron* **2008**, *64*, 3905-3911.

[2] G. S. Hammond, C. E. Reeder, *J. Am. Chem. Soc*. **1958**, *80*, 573-575.

[3] D. P. Curran, A. I. Keller, *J. Am. Chem. Soc*. **2006**, *128*, 13706-13707.

[4] B. Tao, D. W. Boykin, *J. Org. Chem.* **2004**, *69*, 4330-4335.

[5] S. Zhu, S. Shi, S. W. Gerritz, M. J. Sofia, *J. Comb. Chem.* **2003**, *5*, 205-207.

[6] S. B. Waghmode, S. R. Borhade, *Ind. J. Chem*. **2010**, *49B*, 565-572.

[7] Y. Endo, K. Shudo, T. Okamoto, *J. Am. Chem. Soc*., **1982**, *104*, 6393-6397.

[8] D. Badone, M. Baroni, R. Cardamone, A. Ielmini, U. Guzzi, *J. Org. Chem.* **1997**, *62*, 7170-7173.

[9] Q. J. Zhou, K. Worm, R. E. Dolle, *J. Org. Chem.* **2004**, *69*, 5147-5149.

[10] L. Liu, Y. Zhang, Y. Wang, *J. Org. Chem.* **2005**, *70*, 6122-6125.

[11] M. E. Mowery, P. DeShong, *J. Org. Chem*. **1999**, *64*, 3266-3270.

[12] U. Grether, H. Waldmann, *Chem. Eur. J*. **2001**, *7*, 959-971.

[13] B. Tao, D. W. Boykin, *J. Org. Chem*. **2004**, *69*, 4330-4335.

[14] Y. Hatanaka, K. Goda, Y. Okahara, T. Hiyama, *Tetrahedron* **1994**, *50*, 8301-8319.

[15] Y. M. A. Yamada, K. Takeda, H. Takahashi, S. Ikegami, *Org. Lett*. **2002**, *4*, 3371-3374.

[16] S. Paul, J. H. Clark, *Green Chem.* **2003**, *5*, 635-638.

[17] D. J. Sinclair, M. S. Sherburn, *J. Org. Chem*. **2005**, *70*, 3730-3733.
